# Strengthening effects of bone marrow mononuclear cells with intensive atorvastatin in acute myocardial infarction

**DOI:** 10.1136/openhrt-2019-001139

**Published:** 2020-05-10

**Authors:** Yue-Jin Yang, Hai-Yan Qian, Lei Song, Yong-Jian Geng, Run-lin Gao, Na Li, Hong Wang, Xia-Qiu Tian, Ji Huang, Pei-Sen Huang, Jun Xu, Rui Shen, Min-Jie Lu, Shi-Hua Zhao, Wei-Chun Wu, Yuan Wu, Jun Zhang, Jie Qian, Jun-Yan Xu, Yu-Yan Xiong

**Affiliations:** 1 Department of Cardiology, Center for Coronary Heart Disease, State Key Laboratory of Cardiovascular Disease, Fuwai Hospital, National Center for Cardiovascular Diseases, Chinese Academy of Medical Sciences and Peking Union Medical College, Beijing, China; 2 The Center for Cardiovascular Biology and Atherosclerosis, Department of Internal Medicine, University of Texas McGovern School of Medicine at Houston, Houston, Texas, USA; 3 Department of Cardiology, Beijing Chaoyang Hospital, Capital Medical University, Beijing, China; 4 Center for Cardiac Critical Care, Beijing Anzhen Hospital, Capital Medical University, Beijing Institute of Heart Lung and Blood Vessel Diseases, Beijing, China; 5 Department of Cardiology, Beijing Anzhen Hospital, Capital Medical University, Beijing Institute of Heart, Lung and Blood Vessel Diseases, Beijing, China; 6 Department of Nuclear Medicine, Fuwai Hospital, National Center for Cardiovascular Diseases, Chinese Academy of Medical Sciences and Peking Union Medical College, Beijing, China; 7 Department of Magnetic Resonance Imaging, Fuwai Hospital, National Center for Cardiovascular Diseases, Chinese Academy of Medical Sciences and Peking Union Medical College, Beijing, China; 8 Department of Echocardiography, State Key Laboratory of Cardiovascular Disease, Fuwai Hospital, National Center for Cardiovascular Diseases, Chinese Academy of Medical Sciences and Peking Union Medical College, Beijing, China

**Keywords:** acute coronary syndrome, myocardial perfusion, statins

## Abstract

**Objective:**

To test whether intensive atorvastatin (ATV) increases the efficacy of transplantation with autologous bone marrow mononuclear cells (MNCs) in patients suffering from anterior ST-elevated myocardial infarction (STEMI).

**Methods:**

This clinical trial was under a 2×2 factorial design, enrolling 100 STEMI patients, randomly into four groups of regular (RA) or intensive ATV (IA) with MNCs or placebo. The primary endpoint was the change of left ventricular ejection fraction (LVEF) at 1-year follow-up from baseline, primarily assessed by MRI. The secondary endpoints included other parameters of cardiac function, remodelling and regeneration determined by MRI, echocardiography, positron emission tomography (PET) and biomarkers.

**Results:**

All the STEMI patients with transplantation of MNCs showed significantly increased LVEF change values than those with placebo (p=0.01) with only in the IA+MNCs patients group demonstrating significantly elevation of LVEF than in the IA+placebo group (+12.6% (95%CI 10.4 to 19.3) vs +5.0% (95%CI 4.0 to 10.0), p=0.001), pointing to a better synergy between ATV and MNCs (p=0.019). PET analysis revealed significantly increased viable areas of myocardium (p=0.015), while the scar sizes (p=0.026) and blood aminoterminal pro-B-type natriuretic peptide (p<0.034) reduced. All these above benefits of MNCs were also attributed to IA+MNCs instead of RA+MNCs group of patients with STEMI.

**Conclusions:**

Intensive ATV treatment augments the therapeutic efficacy of MNCs in patients with anterior STEMI at the convalescent stage. The treatment with the protocol of intensive ATV and MNC combination offers a clinically essential approach for myocardial infarction.

**Trial registration number:**

NCT00979758.

**Key questions**

**What is already known about this subject?**

- Many clinical trials revealed the potential of bone marrow mononuclear cells (MNCs) therapy for acute myocardial infarction (AMI). However, the limited efficacy of MNCs was not as promising as those from the animal studies, which is the main bottle-neck limitation since the transplanted stem cells barely retain and hardly survive in the infarct area because of the harsh postinfarct microenvironments.
- For improving the effectiveness of stem cells therapy, we have reported that pretreatment with high dose of statins with anti-inflammation and pleiotropic effects on cardiovascular cells can increase survival of implanted MSCs in the infarcted myocardium, accompanied by improvement of left ventricular ejection fraction (LVEF) and myocardial metabolism in porcine AMI models.

**What does this study add?**

- We hypothesised that pretreatment with statins may offer a novel strategy for improving the harsh microenvironment in infracted myocardium and augmenting the efficacy of MNCs transplantation in AMI patients.
- We conducted the trial with a 2×2 factorial design, and enrolled 100 patients with extensive anterior wall ST-elevated myocardial infarction, who were randomly allocated into four groups of regular (RA) or intensive atorvastatin (IA) with MNCs or placebo. The primary endpoint was the change value of LVEF at 1-year follow-up with MRI.
- The change value of LVEF in total MNCs patients was significantly increased compared with that in total placebo patients (p=0.01), with only in IA+MNCs significantly enhanced compared with IA+placebo groups (+12.6% (95%CI 10.4 to 19.3) vs +5.0% (95%CI 4.0 to 10.0), p=0.001) and a significant synergy between atorvastatin and MNCs (p=0.019). Positron emission tomography revealed significant increase in the viable myocardial area (p=0.015) with significant reductions in scar sizes (p=0.026) of MRI and blood aminoterminal pro-B-type natriuretic peptide (p<0.034). All these benefits in total MNCs patients occurred only in IA+MNCs group as well.

**How might this impact on clinical practice?**

- IA in peritransplant period remarkably augments the therapeutic efficacy of MNCs in patients with anterior AMI even at the convalescent stage, thus providing a novel strategy for enhancing the efficacy of MNC transplantation in clinical treatment of AMI patients.

## Introduction

Many clinical trials have shown that transplantation of bone marrow cells may possess the therapeutic benefit for acute myocardial infarction (AMI).^1–5^ Mononuclear cell (MNC) transplantation reportedly promotes myocardial repair.^6 7^ However, there is relatively limited improvement of left ventricular ejection fraction (LVEF), which is not as promising as those from the animal studies.^8 9^ The main bottle-neck limitations may be that few transplanted stem cells survive the harsh microenvironments of the infarcted cardiac tissue.^10^

In order to improve the effectiveness of stem cell therapy, we have previously explored the possibility that preconditioning the ischaemic myocardium with high doses of statins exerts pleiotropic effects on cardiovascular cells.^10 11^ Our previous studies in porcine AMI models have demonstrated that intensive statin administration increases the potency of implanted MSCs for survival, repair and regeneration in the infarcted myocardium, with improved LVEF and myocardial metabolism^11 12^ through reducing the harshness of microenvironments which contain inflammatory and cytotoxic substances, that is, ‘fertilising the poor soil’ at the infarcted region. Our recent work has also shown that the intensive statin treatment activates endothelial nitric oxide synthase.^12^ Interestingly, our previous study has shown that treatment with statins promotes the cardiomyogenesis of stem cells via inducing expression of promyogenic genes.^13^

Therefore, we have pursued a clinical study to translate the preclinical research into the combined application of intensive statin and MNCs in patients with ST-elevated myocardial infarction (STEMI) to offer a novel strategy for improving the postinfarct cardiac microenvironment and augmenting the efficacy of MNC transplantation in AMI patients.

## Methods

### Participants

A total of 100 STEMI patients were enrolled into a phase-II, randomised, double-blind, placebo-controlled trial in a single-centre between January 2009 and February 2014. The inclusion criteria were as follows: (1) ages between 30 and 80 years; (2) 2–4 weeks after the onset STEMI in the left ventricular anterior wall; (3) evidence of non-viable infarcts by positron emission tomography (PET) (4) LVEF ≤45% by two-dimensional echocardiography (2DE). The exclusion criteria are as follows: (1) non-STEMI (NSTEMI), (2) LVEF >45%, (3) severe valve heart disease or mechanical complications including ventricular septal perforation, tendon or papillary muscle rupture, (4) other systemic diseases including acute infectious diseases, haematologic diseases, severe kidney dysfunction, serious liver damage, unstable brain lesions, malignant tumours, pregnancy and severe mental or physical disability who will not be able to follow-up.

### Study design and procedures

**Figure 1:**
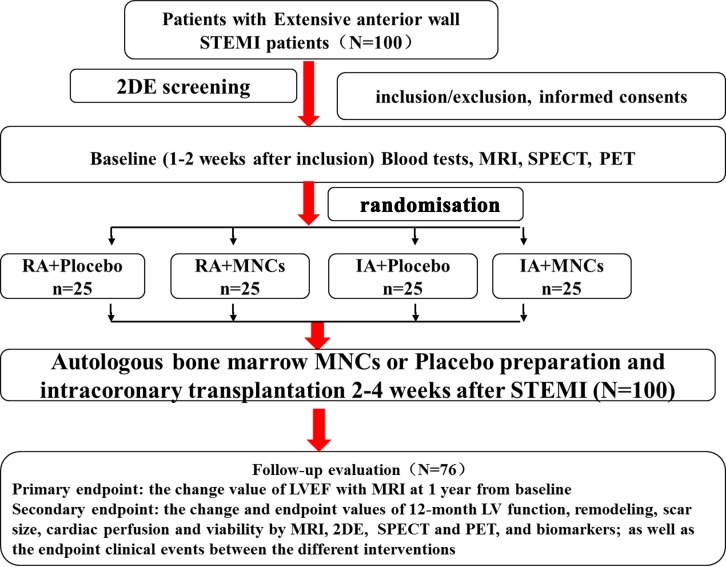
Schematic representation of the study design and grouping. 2DE, two-dimensional echocardiography; IA, intensive atorvastatin; LVEF, left ventricular ejection fraction; MNCs, mononuclear cells; PET, positron emission tomography; RA, routine atorvastatin; SPECT, single-photon emission CT; STEMI, ST-segment elevation myocardial infarction.

The trial was conducted following a 2×2 factorial design. By applying a web-based automated random number generator as previously described,^11 12^ a treatment schedule will be prepared by a designated researcher who will have no contact with any participants, and patients at the convalescent stage (2–4 weeks post-STEMI) were randomly allocated to treatment with regular (RA, 20 mg/day) or intensive (IA, 80 mg/day) atorvastatin (ATV, Lipitor, Pfizer Pharmaceutical Company) for 3 days, and then to intracoronary infusion with autologous bone marrow-derived MNCs or placebo. In essence, patients were divided into four groups (25 each) of regular atorvastatin with placebo (RA+placebo) or MNCs (RA +MNCs), and intensive atorvastatin (IA) with placebo (IA+placebo) or MNCs (IA+MNCs). After MNCs transplantation, only patients in the two IA groups received 4 days more of IA then followed by regular atorvastatin as in the two RA groups with clinical followed-up and evaluation for up to 1 year. All the investigators, including clinical and imaging professionals, and the patients were blinded to the information of treatment and grouping. Furthermore, the tests and data processing of MRI, 2DE, PET with single-photon emission CT (SPECT) were, respectively, and independently performed by the designated doctors in the each core laboratory who were also blinded to the information (figure 1).

### Primary and secondary endpoints

The primary endpoint was the change value of LVEF from the baseline to the end of 1-year follow-up. The secondary endpoints included the change and endpoint values of other functional and morphological parameters and infarct scar sizes as measured by 2DE and MRI, myocardial perfusion and viability by SPECT and PET, and the cardiac biomarkers in the blood, including aminoterminal pro-B-type natriuretic peptide (NT-proBNP), high sensitivity C reactive protein (hs-CRP) and endothelin. Clinical events were monitored closely, including cardiac death, myocardial reinfarction, repeated revascularisation and malignant ventricular arrhythmias, such as ventricular tachycardia, flutter or fibrillation.

### Preparation and intracoronary infusion of autologous bone marrow MNCs

Following the protocol reported previously by our team and others,^11 12 14^ MNCs were prepared from the bone marrow aspirated from the posterior iliac crest under local anaesthesia. The preparation of MNCs or placebo in detail was described in the online supplementary data part 1. Patients who underwent the primary percutaneous coronary intervention were examined by coronary angiography and then MNCs or placebo infusion was performed. Otherwise, patients were first revascularised for the infarct-related coronary artery left anterior descending and then given by the MNCs infusion following the index procedure. The stem cell delivery in detail was described in the online supplementary data part 2.

10.1136/openhrt-2019-001139.supp1 Supplementary data

### Cardiac MRI

The MRI examination and analysis in detail were described in the online supplementary data part 3.

### Two-dimensional echocardiography (2DE)

The 2DE examination and analysis in detail were described in the online supplementary data part 4.

### Analysis of myocardial perfusion and viability by SPECT and PET

Myocardial perfusion was assessed by a dual-head SPECT (Siemens Medical, GER) 90 min after intravenously injecting a dose of 740 MBq technetium-99m sestamibi (99mTc-MIBI), and PET scans were performed within 2 days after SPECT (the process of examination and analysis in detail were described in the online supplementary data part 5).

### Analysis of blood biomarkers

The NT-proBNP and endothelin-1 were analysed using an immunoassay according to manufacturer’s instructions (Roche Elecys, Roche Diagnostics, Basel, Switzerland; R&D Systems, Minneapolis, MN, USA). The hs-CRP concentrations were measured with an automated chemistry analyser using commercial kits (Abbott Aeroset, Minnesota, USA).

### Safety monitoring

All the participants were monitored for adverse events and the associated information would be transmitted to the Data and Safety Monitoring Board, the Institutional Review Board of our centre and China Food and Drug Administration (CFDA). The trial stopping rules were developed in consultation with the CFDA.

### Statistical analyses and sample size calculation

The clinical trial was designed using a 2×2 factorial model in which intervention effectiveness was compared between patients treated with statin-enhanced and control MNCs. Patients with implantation of MNCs were compared when they received regular or intensive statin therapy. Such comparisons were performed in a multivariable regression model, including the interventions, corresponding interactions as well as the values of outcome variables. Power analysis was performed to determine the sample sizes with the statistical significance level taken as one-sided 0.025 and the power as 80%. The quantitative data were presented as mean±SD. Categorical data were presented as frequencies and percentages. Continuous variables at baseline with those at follow-up were compared with the paired analysis of variance. Continuous variables with a non-normal distribution between groups were compared using the Wilcoxon rank sum test and the Mann-Whitney test. Statistical significance was set at p<0.05. Data were analysed using SPSS V.19.0 (online supplementary data part 6).

## Results

All the 100 participants enrolled in this study received the baseline assessments. Among them, 76 patients did completion of the 1-year end-point follow-up assessments and their data entered the final analysis. The remaining 24 participants who did not complete the 1-year endpoint evaluations due to geological distance and personal inconvenience were excluded from final analysis. Comparison in the baseline characteristics between the patients with and without completion of final assessments indicated no significant difference (online supplementary table 1). In addition, no significant differences in the baseline characteristics and all the parameters of coronary lesions and interventions occurred between the four groups or under RA and IA groups (table 1 and online supplementary table 2).

**Table 1:** Baseline characteristics of the four groups

	RA+placebo (n=19)	RA+MNCs (n=20)	IA+placebo (n=17)	IA+MNCs (n=20)	P value
Male (%)	18 (94.7)	18 (90.0)	15 (88.2)	17 (85.0)	0.797
Age (years)	51.7±9.1	57.0±12.7	50.1±13.0	52.9±13.8	0.360
BMI (kg/m^2^)	25.5±6.2	25.2±3.0	26.6±4.6	26.2±3.0	0.773
Killip class	1.89±1.29	1.60±0.88	1.53±0.87	1.75±1.07	0.720
IABP (%)	3 (15.8)	1 (5.0)	1 (5.9)	4 (20.0)	0.386
Hypertension (%)	10 (52.6)	12 (60.0)	7 (41.2)	9 (45)	0.668
Diabetes (%)	6 (31.6)	4 (20.0)	4 (23.5)	3 (15.0)	0.666
Hyperlipidaemia (%)	8 (42.1)	3 (15.0)	8 (47.1)	7 (35.0)	0.168
Stroke (%)	0	0	0	1 (5.0%)	0.417
Smoking (%)	17 (89.5)	13 (65.0)	13 (76.5)	10 (50.0)	0.050
CHD family history (%)	4 (21.1)	2 (10.0)	3 (17.6)	2 (10.0)	0.729
Previous MI (%)	2 (10.5)	4 (20%)	0 (0)	0 (0)	0.055
Previous coronary revascularisation (%)	1 (5.3)	2 (10.0)	0 (0)	0 (0)	0.503
SBP (mm Hg)	113.1±16.0	110.7±13.0	120.8±17.6	115.2±19.2	0.307
HR (bpm)	80.6±19.2	75.8±14.8	76.3±10.9	82.1±16.9	0.520
ALT (IU/L)	86.4±76.1	57.0±41.9	46.2±41.2	56.4±32.8	0.100
Scr (umol/L)	84.2±10.9	89.0±19.7	87.5±17.0	85.9±14.7	0.804
HbA1c (%)	6.4±1.2	6.5±1.4	6.6±1.5	6.2±1.3	0.866
WBC (*10^9^/L)	9.9±3.5	9.8±5.5	8.8±2.4	9.0±4.3	0.821
HCT (%)	42.3±5.0	43.3±4.5	39.9±3.7	39.5±8.9	0.155
HGB (g/L)	140.1±19.3	145.6±16.3	138.3±10.0	136.6±14.1	0.295
PLT (*10^9^/L)	262.8±119.9	226.3±92.1	258.7±93.0	252.4±83.0	0.656
Medications					
Aspirin (%)	19 (100)	20 (100)	17 (100)	20 (100)	NS
Clopidogrel (%)	19 (100)	20 (100)	17 (100)	20 (100)	NS
Beta-blockers (%)	17 (89.5)	19 (95.0)	16 (94.1)	18 (90.0)	0.912
RAASi (%)	14 (73.7)	15 (75.0)	13 (76.5)	14 (70.0)	0.844
Spirolactone (%)	13 (68.4)	12 (60.0)	11 (64.7)	14 (70.0)	0.638
Diuretics (%)	15 (78.9)	14 (70.0)	13 (76.5)	13 (65.0)	0.597

ALT, alanine aminotransferase; BMI, body mass index; CHD, coronary heart disease; HbA1c, glycosylated haemoglobin; HCT, haematocrit; HGB, haemoglobin; HR, heart rate; IA, intensive aotrvastain; IABP, intra-aortic balloon pump; LDL-C, low-density lipoprotein cholesterol; MI, myocardial infarction; MNCs, mononuclear cells; NS, not significant; PLT, platelet; RA, regular atorvastatin; RAASi, renin angiotensin aldosterone system inhibitor; SBP, systolic blood pressure; Scr, serum creatinine; WBC, white blood cell.

The analysis of the blood lipid profile parameters from baseline to endpoint showed that the decrease in LDL-C levels was significantly more only in IA treatment patients than that in RA patients (−0.78 mmol/L(−0.97 to –0.59) vs −0.32 mmol/L (−0.51 to –0.18), p=0.013) (online supplementary table 3).

### MRI assessment of cardiac performance, remodelling and scar sizes

**Figure 2:**
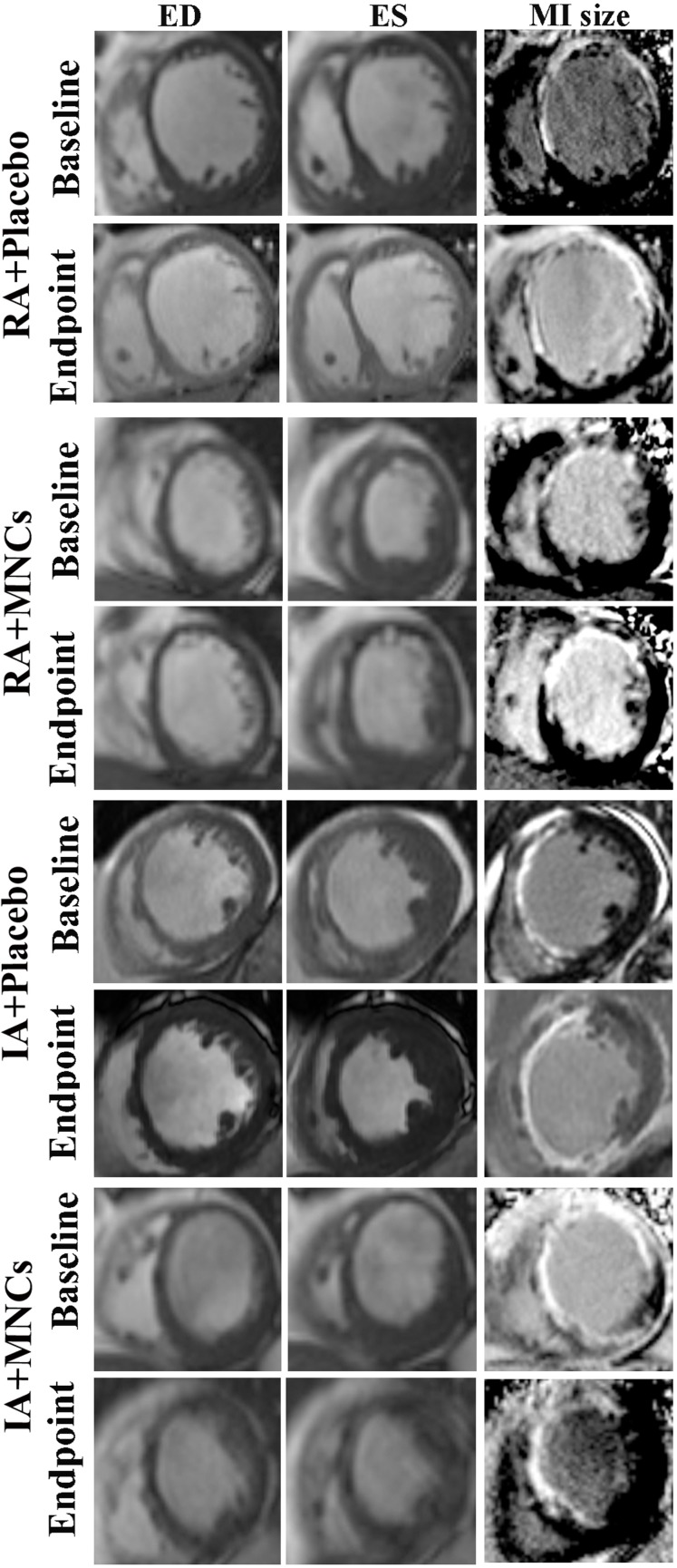
Representative MRI images of cardiac left ventricle in myocardial infarct (MI) patients treated with intensive (IA) or regular atorvastatin (RA) in combination with or without transplantation of mononuclear cells (MNCs). MRI was conducted at short axis in the end-diastolic (ED) or end-systolic (ES) phase.

As shown in figure 2 and table 2A, B the change value of LVEF from baseline to endpoint in total MNCs group was significantly increased compared with that in total placebo group (+10.0% (95% CI 8.0 to 12.6) vs +5.0% (95% CI 3.5 to 9.2), p=0.010). However, the significant changes were observed only between IA intragroups (+12.6% (95% CI 10.4 to 19.3) vs +5.0% (95% CI 4.0 to 10.0), p=0.001) but not between RA intragroups (p=0.809). There was a significantly synergistic improvement in LVEF between atorvastatin and MNCs (p=0.019). The endpoint value of LVEF in total MNCs group was also significantly increased compared with that in total placebo group (46.0% (95% CI 44.0 to 49.5) vs 39.5% (95% CI 37.0 to 43.3), p=0.009), with only in IA+MNCs being significantly higher than in IA+placebo groups as well (47.5% (95% CI 45.0 to 52.6) vs 38.0% (95% CI 34.0 to 41.0), p=0.001).

**Table 2:** (A) Left ventricular function, remodelling and scar size assessment by MRI. (B) Comparison of MRI parameters between ATV groups

A	Total RA (n=39)	Total IA (n=37)	Total placebo (n=36)	Total MNCs (n=40)
LVEF				
Baseline	36.0 (33.0,39.4)	32.0 (31.0,34.4)	33.5 (31.5,35.0)	34.2 (32.7,36.5)
Endpoint	44.0 (41.0,46.5)	43.0 (39.0,46.0)	39.5 (37.0,43.3)	46.0 (44.0,49.5)*
Adjusted difference† (95% CI)	6.9 (4.0 to 9.0)	10.0 (7.6 to 12.1)	5.0 (3.5 to 9.2)	10.0 (8.0 to 12.6)
P value	0.089		0.01	
EDVi (mL/m^2^)				
Baseline	81.5 (74.4,89.7)	82.2 (75.0,91.0)	81.9 (74.4,89.7)	81.8 (73.2,90.0)
Endpoint	67.9 (62.0,75.6)	71.0 (63.7,81.9)	72.3 (62.5,78.1)	68.5 (64.6,78.3)
Adjusted difference† (95% CI)	−7.8 (–18.8 to –3.0)	−21.4 (–24.4 to –3.2)	−6.9 (–17.1 to –1.7)	−9.2 (–18.0 to –0.9)
P value	0.718		0.832	
ESVi (mL/m^2^)				
Baseline	49.8 (46.6,61.0)	55.7 (51.0,61.0)	50.4 (47.0,60.5)	55.6 (49.3,62.4)
Endpoint	39.0 (32.0,44.0)	37.0 (32.0,45.0)	37.0 (32.5,45.0)	39.0 (32.0,44.2)
Adjusted difference† (95% CI)	−9.7 (–12.2 to –0.8)	−11.2 (–29.9 to –0.7)	−8.1 (–21.4 to –1.2)	−18.5 (–26.3 to –6.1)
P value	0.533		0.234	
Scar size (mm3)				
Baseline	39.2 (31.8,42.0)	41.5 (33.0,44.8)	39.2 (32.4,43.9)	40.0 (32.7,42.2)
Endpoint	40.2 (35.7,44.5)	35.9 (33.0,43.8)	38.6 (34.6,50.1)	36.5 (31.0,42.0)‡
Adjusted difference† (95% CI)	1.67 (–0.16 to 4.80)	0.81 (–3.32 to 2.62)	2.60 (1.18 to 6.94)	−2.65 (–5.10 to 1.60)
P value	0.659		0.01	

B	Total RA (n=39)		Total IA (n=37)	
	RA+placebo (n=19)	RA+MNCs (n=20)	IA+placebo (n=17)	IA+MNCs (n=20)
LVEF				
Baseline	37.8 (29.7,40.7)	35.5 (33.0,38.8)	32.5 (30.4,34.7)	31.6 (29.0,36.0)
Endpoint	43.6 (37.0,46.5)	45.5 (41.1,49.5)	38.0 (34.0,41.0)	47.5 (45.0,52.6)§
Adjusted difference† (95% CI)	3.4 (0.3 to 12.6)	7.8 (6.3 to 10.0)	5.0 (4.0 to 10.0)	12.6 (10.4 to 19.3)
P value	0.809		0.001	
EDVi (mL/m^2^)				
Baseline	81.5 (74.4,92.1)	82.5 (67.5,92.7)	82.2 (73.5,101.0)	81.8 (73.2,92.6)
Endpoint	71.8 (61.1,75.6)	67.1 (61.0,79.4)	76.0 (59.6,94.0)	70.3 (65.0,81.9)
Adjusted difference† (95% CI)	−3.3 (–13.2 to 0.8)	−11.5 (–25.4 to –2.4)	−8.3 (–18.6 to 4.4)	−8.4 (–16.4 to –0.19)
P value	0.799		0.987	
ESVi (mL/m^2^)				
Baseline	49.1 (45.0,63.0)	52.9 (41.0,65.5)	54.1 (43.7,61.0)	56.0 (49.5,67.4)
Endpoint	36.5 (31.0,47.0)	39.1 (29.5,44.1)	37.0 (29.2,61.0)	38.0 (30.2,47.9)
Adjusted difference† (95% CI)	−4.5 (−8.5 to 0.7)	−17.5 (−27.8 to –6.1)	−21.4 (−24.3 to 1.4)	−21.1 (−30.1 to –2.0)
P value	0.556		0.290	
Scar size (mm^3^)				
Baseline	39.3 (30.5,47.9)	39.1 (30.3,45.4)	33.8 (31.8,47.8)	41.6 (32.3,49.7)
Endpoint	40.2 (33.0,51.1)	40.0 (31.4,43.7)	36.8 (33.8,55.4)	34.6 (28.0,41.2)
Adjusted difference† (95% CI)	2.06 (0.64 to 8.13)	−1.18 (−4.64 to 3.77)	3.06 (0.81 to 11.59)	−3.13 (−5.63 to 1.13)
P value	0.161		0.026	

*Endpoint values in total MNCs group vs total placebo group (p=0.009).

†Values are median (95% CI), and adjusted for values at baseline and the other intervention.

‡Endpoint values in total MNCs group vs total placebo group (p=0.008).

§The comparison of endpoint values in IA+ MNCs group vs IA+placebo group (p=0.001).

ATV, atorvastatin; EDVi, end-diastolic volume index; ESVi, end-systolic volume index; IA, intensive atorvastatin; LVEF, left ventricular ejection fraction; MNCs, mononuclear cells; RA, regular atorvastatin.

Similarly, the MNCs implantation significantly reduced the scar sizes of MRI compared with placebo (p=0.010), with only in patients receiving intensive instead of regular atorvastatin reaching significance, too (p=0.026). No synergistic effect was detected between intensive ATV and MNCs therapy (p=0.572).

In order to evaluate the impacts of the missing data (lost to follow-up), we also added a sensitivity analysis on the primary endpoint (changes on LVEF between baseline and 1-year follow-up) according to the intention-to-treat principle. Last observation carried forward strategy had been used to impute the missing data of LVEF. Because the 24 patients did not have any LVEF data measured with MRI during their follow-up period, the baseline values were used for the imputation. After the ‘missing LVEF values’ of the 24 patients were added in the final analyses, the results were consistent with those achieved from only 76 patients who completed the endpoint examinations (online supplementary table 4).

### Analysis of cardiac function and remodelling by echocardiography

As shown in online supplementary figure 1 and online supplementary table 5, as with MRI evaluation, 2DE demonstrated significant change values of LVEF from baseline to endpoint in total MNCs group and only in IA+MNCs group than total placebo group (+9%(5,10) vs +4%(2,6); p=0.022) and IA+placebo group (+10%(8,12) vs +4% (0,10); p=0.045), with no significant synergy between atorvastatin treatment and MNC implantation (p=0.594).

Consistently, the change values of both EDV and ESV significantly decreased in total MNCs group compared with those in total placebo group (p=0.041 and p=0.03), with both of the change values being significantly reduced only in IA+MNCs compared with IA+placebo groups (p=0.014 and p=0.020), with no significant synergy between atorvastatin and MNCs (p=0.217 and p=0.444).

### Assessment of myocardial perfusion and viability by SPECT and PET

**Figure 3:**
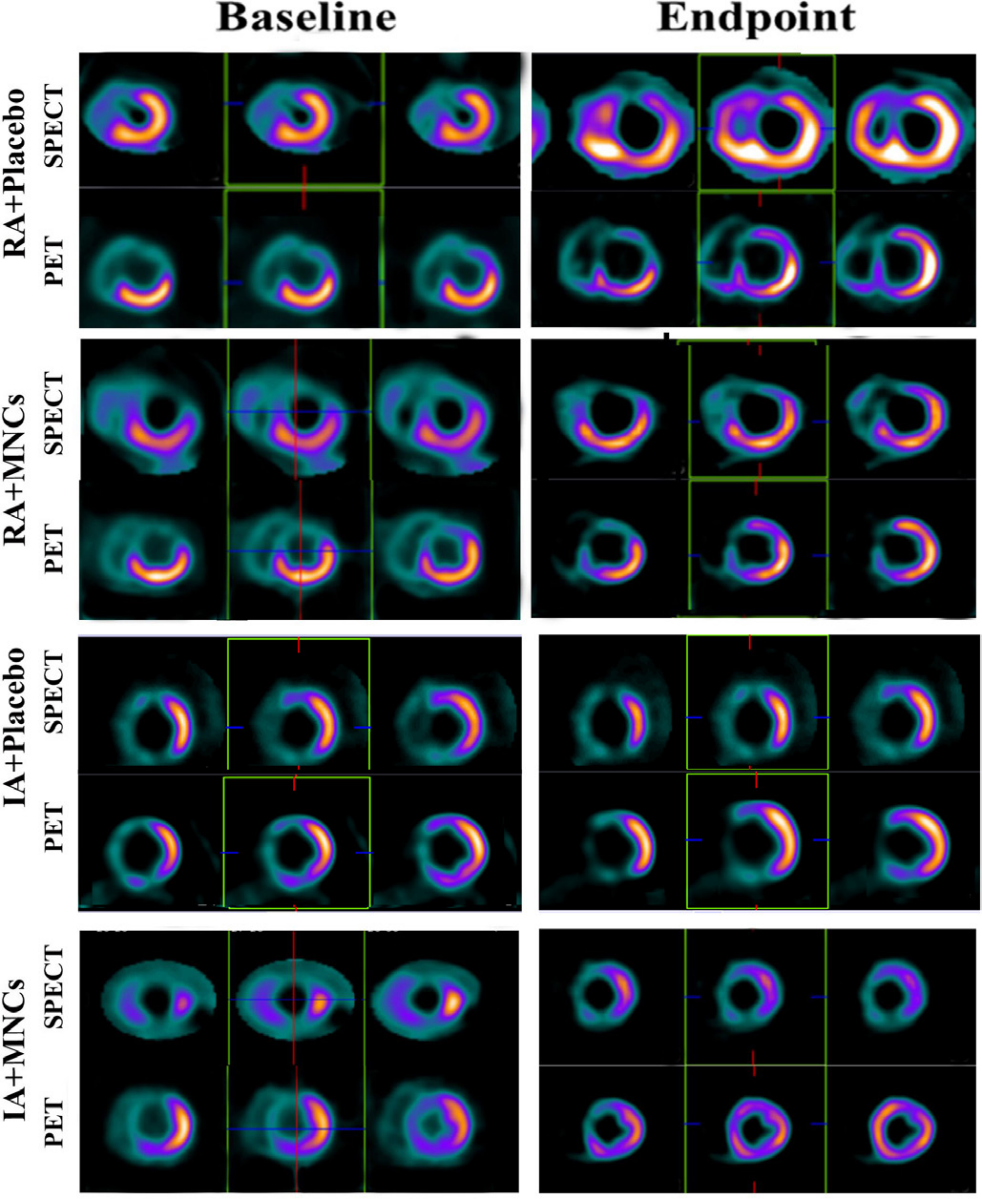
The representative images of SPECT and PET denoting myocardial regeneration after combined treatment with intensive atorvastatin (IA) and MNCs in the same patient shown in figure 2. A, intensive atorvastatin; MNCs, mononuclear cells; PET, positron emission tomography; RA, regular atorvastatin; SPECT, single-photon emission CT

The change values of both perfusion defect sizes and summed rest score were not significantly different between the total MNCs and placebo groups, or between IA and RA groups (online supplementary table 6). However, the change value of FDG score in total MNCs were significantly increased compared with that in total placebo groups (p=0.017), and this benefit was observed only in IA+MNCs compared with IA+placebo group (p=0.015), indicating possible myocardial regeneration occurred in the IA+MNCs patients. No synergistic effect was observed between atorvastatin and MNCs though (p=0.081) (figure 3)

### Changes in blood biomarkers

**Figure 4:**
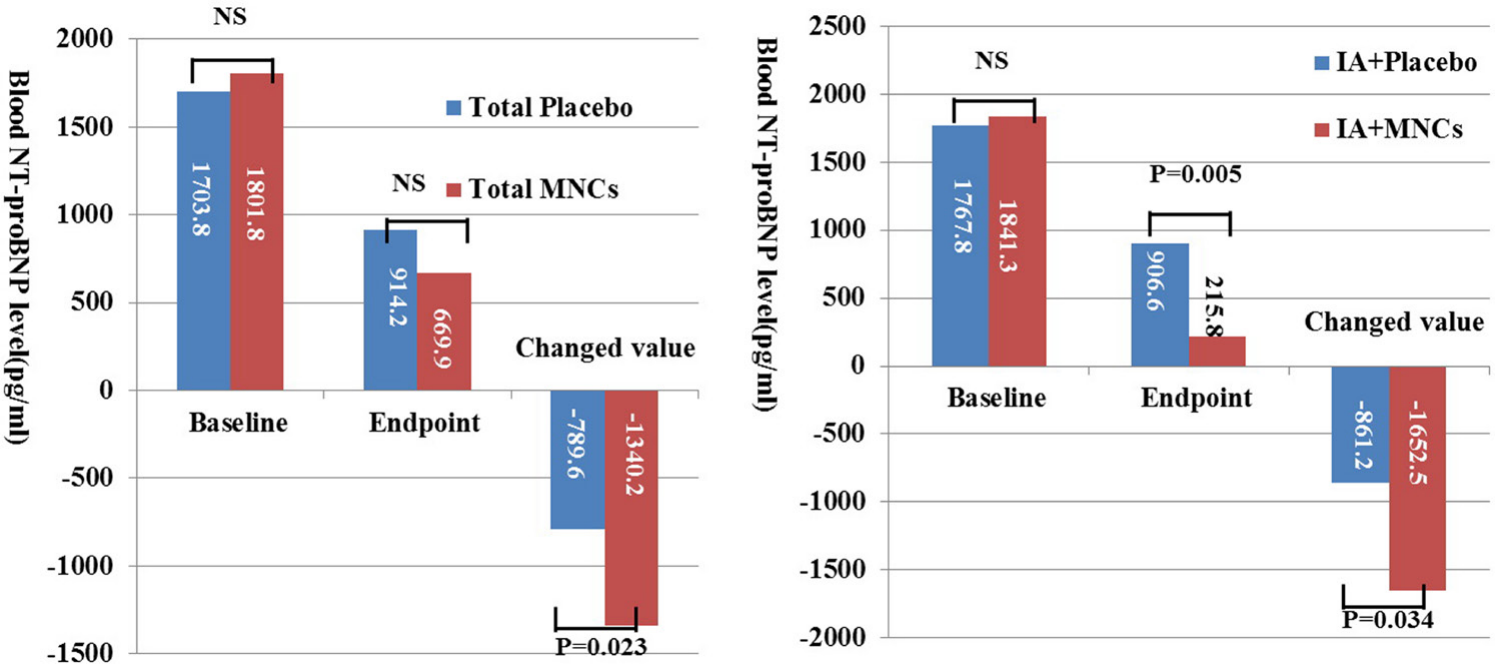
Changes in the blood levels of NT-proBNP after MNC transplantation with or without intensive ATV (IA). ATV, atorvastatin; MNCs, mononuclear cells; proBNP, pro-B-type natriuretic peptide; NS, not significant.

The change values of blood NT-proBNP from baseline to endpoint in the total MNCs patients were significantly higher than that in total placebo patients (p=0.023), with both change and endpoint values significantly increased and decreased respectively, only between IA subgroups (p=0.034 and p=0.005, respectively), indicating that IA treatment improved the left ventricular function evaluated with specific biomarkers as well. There were no significant synergy between atorvastatin treatment and MNCs therapy (p=0.165). Meanwhile, the endpoint value of hs-CRP was significantly lower only in total IA than RA groups (p=0.001), and the change value of endothelin was significantly higher only in IA+MNCs than in IA+placebo groups as well (p=0.046) (figure 4 and online supplementary table 7).

### Assessment of safety

The safety of intracoronary MNCs infusion was evidenced by the following observations throughout the treatment and follow-up period: (i) no death or recurrent MI in patients receiving treatments; (ii) no target lesion revascularisation; no new arrhythmia, for example, sustained ventricular tachycardia or fibrillation. All the enrolled patients including 24 patients who did not finish the endpoint instrumental examinations completed telephone follow-up survey, and they all were alive to the moment of current report (online supplementary table 8).

## Discussion

The present study provides the first clinical evidence that intensive statins apparently improves the therapeutic effects of MNCs transplantation in terms of LVEF elevation for patients with extensive anterior STEMI at the convalescent stage. The main benefits included (1) enhancement of cardiac performance with LVEF value increasing by 10% or even higher; (2) attenuation of LV remodelling; (3) decrease of scar size with the increase in the area of myocardial viability; (4) reduction in blood NT-pro BNP levels.

With a mixture of multiple types of cells, the bone marrow MNCs provide the ecosystem that comprises various cell lineages, including haematopoietic progenitor cells, endothelial progenitor cells, stromal cells and others, bearing characteristics of plasticity and availability.^14^ The positive results from several early clinical trials have shown that intracoronary infusion of bone marrow MNCs in AMI patients has therapeutic effects, such as the left ventricular function improvement and attenuation of negative remodelling.^15 16^ However, the reported efficacy is very low with LVEF elevation by only 2.5%–3.9%,^6 7^ and even negative in later clinical trials.^9 17 18^ The low rates of survival of implanted MNCs in infarct area are considered to be the main factors to limit the effectiveness of the stem cell therapy because of bad microenvironment in the infarct myocardium. The present clinical trial is the first to demonstrate that pretreatment with intensive oral statin markedly augments the efficacy of MNCs therapy with LVEF elevating by 10% or even more, since it improves the harsh microenvironment of infarct region as like ‘fertilisation of barren land’. Our observations suggest that intensive ATV preconditions is the prerequisite in stem cell therapy. This benefit provided with the combination therapy of intensive statin and MNCs is much more than that from previous clinical studies of MNCs alone.^7 11 12^ The synergistic effects between intensive ATV and MNCs shown in the clinical trial are consistent with our previous reports in the porcine model in vivo as well.^10 12 19^ To the best of our knowledge, this is the first clinical report that the combination protocol of bone marrow MNCs with intensive ATV has achieved such an impressive efficacy in patients with STEMI, denoting the essential role of ‘fertilisation’ with intensive ATV in clinical stem cell therapy.

The synergism between intensive ATV and MNC transplantation is supported by several cellular and molecular biomarkers in the hearts. Apart from the anti-inflammatory and antiapoptotic effects by MSCs themselves, the intensive ATV treatment likely improves the microenvironment via the pleiotropic effects, such as the endothelial protection, anti-inflammation, antioxidative stress and antiapoptosis for ‘fertilising the poor soil’ in infarct area^11 12 19 20^ and then enhances the survival and angiogenesis of implanted MNCs in addition to upregulating of stromal derived factor-1 in infarct myocardial for MSCs directional homing.^21–25^ The combined treatment with intensive statin and MNCs reduces the scar size, which is also consistently with the results from recent animal studies reported by our team.^25 26^

Moreover, the 2DE data obtained in the present study have shown that the intensive but regular ATV combined with MNC therapy can also reduce EDV and ESV, supporting an attenuation of left ventricular remodelling. This is consistent with those reported previously in other clinical studies.^1–5^ The mechanism for the ventricular remodelling attenuation with intensive instead of regular dose of atorvastatin remains unclear. Probably its stronger pleiotropic effects, including the lipid lowering and antiapoptotic action, contribute to the improvement.

Another interesting finding of this clinical trial is that the intensive but regular ATV combined with MNCs therapy can increase the area of viable myocardium at infarct region with reduction in scar size and blood level of heart failure biomarker NT-Pro BNP, suggesting the possibility of cardiac tissue repair and regeneration. It is consistent with our previous reports of animal experiments,^10 12 19^ though has not been reported by the other clinical trial on AMI patients receiving MNCs.^27^ The myocardial regeneration in patients received the combined therapy with intensive ATV and MNCs transplantation may be, at least in part, attributed to activation and mobilisation of endogenous stem cells in the peri-infarct regions by MSCs.^28^ However, more vigorous clinical evaluation is needed to obtain further evidence.

The optimal timing for stem cell transplantation remains a controversial issue. The majority of clinical trials with MNCs have applied the cell transplantation during the early period of time (usually within 7 days) following AMI, as reported in the BOOST and REPAIR-AMI trials.^2 4^ The natural expression of SDF-1 in infarct myocardium increases rapidly in 1 to 3 days post-AMI, and decline to the normal level within 1 week.^29^ From a clinical point of view, however, the early intracoronary infusion of MNCs for the critically ill patients is risky due to very low LVEF, dedicated unstable haemodynamics and complications of concomitant heart failure, etc. In the above dilemmatic situations, our current study choose delayed time points (average 4 weeks after STEMI) in intracoronary infusion of MNCs for the patients’ safety. MNCs implantation at the convalescent phase of STEMI in this study results in an improvement of the efficacy with the protocol combined intensive ATV with MNC therapy, which is consistent with the preclinical data,^30^ though to some degrees, inconsistent with the majority of clinical trials.^6 9^ The underlying mechanisms are still unknown, possibly due to the composite effectiveness which includes the above-mentioned synergistic effects, the subsided harsh microenvironment of inflammation, and the remaining SDF-1 levels in the infarct myocardium, the increased CXCR4 expressions in endothelial progenitor cells,^20^ and the target migration of MSCs via the activating SDF-1/CXCR4 axis^23 24^ by intensive statins.

In conclusion, the pretreatment with IA augments the efficacy of MNC transplantation in enhancing the cardiac performance, attenuating ventricular remodelling for patients with STEMI at the convalescent stage, probably through improving the harsh microenvironment in infarct zone and increasing implanted MNC survival and possible regeneration, revealing its essential role in clinical stem cell therapy.
