# Supplementary material for: Strengthening effects of bone marrow mononuclear cells with intensive atorvastatin in acute myocardial infarction
Source: Open Heart. 2020 May 10;7(1):e001139. doi: 10.1136/openhrt-2019-001139 (PMC7223465; doi:10.1136/openhrt-2019-001139)
Supplement: Supplementary data [file openhrt-2019-001139supp001.pdf]

Contents

Supplemental Data Part 1: The preparation of MNCs or Placebo…………..…….P2

Supplemental Data Part 2: Intracoronary infusion of autologous bone marrow MNCs……………………………………………………………………….……..P3

Supplemental Data Part 3: Cardiac MRI………………...………………..…….P4

Supplemental Data Part 4: Two-dimensional echocardiography…………...…….P5

Supplemental Data Part 5: Analysis of myocardial perfusion and viability by SPECT and PET…………………………………………………………..…….P6

Supplemental Data Part 6: Sample size calculation……………………………….P8

Supplemental Figure 1: The reductions in LV volumes with 2DE after MNCs transplants denoting LV remodeling attenuation…………………………………...P10

Supplemental Table 1. Baseline comparison between 24 patients lost to follow-up and 76 ones completing endpoint assessments…………………….……..….P11

Supplemental Table 2: The properties of coronary lesions and intervention…………………………………………………………......…..P13

Supplemental Table 3: Blood lipid profile at baseline and one-year endpoint……………………………………………………………………......P14

Supplemental Table 4: Left ventricular ejection fraction by MRI after adding missing data…………………………………………………………………...….P16

Supplemental Table 5: The structural and functional changes of left ventricle with 2DE after MNCs therapy…………………………..……..………………..….P17

Supplemental Table 6: Myocardial perfusion with SPECT after MNCs transplantation…………………………………………………………………P19

Supplemental Table 7: Biomarkers at baseline and endpoint……………….…….…P20

Supplemental Table 8: MACEs after MNC therapy..…………………….……..…P22

Supplemental References: ……………………………………………….….P23

Yang Y-J, *et al. Open Heart* 2020; 7:e001139. doi: 10.1136/openhrt-2019-001139

Supplemental Data Part 1: The preparation of MNCs or Placebo

As reported previously by our team and others [1-4], MNCs were prepared from the bone marrow aspirated from the posterior iliac crest under local anesthesia. A volume of approximately 80-100ml of bone marrow was harvested aseptically at the day of intracoronary infusion and treated with 10IU/ml of heparin.

Volume depletion and enrichment of bone marrow-derived MNCs were performed using apheresis (Cobe Spectra, Gambro BTC Inc., Lakewood, CO) to obtain a concentrated stem cell suspension. The bone marrow cell preparation was diluted (1:10) with acid citrate dextrose solution A to prevent coagulation. One hour prior to intracoronary infusion, the MNC suspension was centrifuged, and cell pallets were re-suspended in 10 ml heparinized aline. MNCs were examined microscopically and counted by hemocytometer. The same volume of heparinized normal saline was prepared as the placebo for intracoronary infusion.

Yang Y-J, *et al. Open Heart* 2020; 7:e001139. doi: 10.1136/openhrt-2019-001139

Supplemental Data Part 2: Intracoronary infusion of autologous bone marrow MNC

Patients who underwent the primary percutaneous coronary intervention (PCI) were examined by coronary angiography and then MNCs infusion was performed. Otherwise, patients were first revascularizedfor the infarct related coronary artery left anterior descending (LAD) and then given by the MNC infusion following the index procedure. An over-the-wire balloon catheter (Marverick, Boston Scientific Co.) connected with pressure pump containing the MNC solutions was used for stem cell delivery. Intracoronary nitroglycerin was administered to prevent coronary spasms or abrupt closure caused by the high viscosity infusion of the cell solutions. During the infusion, the balloon was inflated for two minutes to prevent washout of MNCs. The procedure of intracoronary cell infusion was well tolerated, and did not show any severe adverse events or increases in the troponin levels. In the placebo, 10 ml heparinized saline solution was infused under the same procedure.

Yang Y-J, *et al. Open Heart* 2020; 7:e001139. doi: 10.1136/openhrt-2019-001139

Supplemental Data Part3: Cardiac MRI

Cardiac MRI was performed using a 1.5-T clinical MRI scanner (Siemen Avanto, Germany) with a phase array radiofrequency receiver coil, electrocardiography gating, and respiratory triggering. Left ventricular (LV) volume and function were evaluated, including LV end-diastolic volume (EDV), end-systolic volume (ESV), and ejection fraction (LVEF). The endocardial and epicardial borders were traced in all end-diastolic and end-systolic short axis and long axis slices to determine EDV and ESV for calculation of global LVEF, with LVEF = [(EDV- ESV)/EDV] × 100%. Two experienced MRI experts blinded to all clinical data analyzed the images. All MRI was quantitatively analyzed according to the previous study of our team and others [2,3,5].

Yang Y-J, *et al. Open Heart* 2020; 7:e001139. doi: 10.1136/openhrt-2019-001139

Supplemental Data Part 4: Two-dimensional echocardiography

Following the previous study protocol[6], 2DE was conducted in the left lateral decubitus position using a clinically available system (Vivid 7 and E9, General Electric-Vingmed, Horten, Norway). Data acquisition was performed with a 3.5-MHz transducer at a depth of 16 cm in the standard parasternal and apical views. During the breath hold, M-mode and two dimension (2D) images were obtained and saved in cine-loop format. Data analysis was performed offline (EchoPAC version 111.0.0; General Electric-Vingmed,). EDV, ESV, and LVEF were derived from the conventional 2D apical two and four-chamber view images, using the biplane Simpson's rule.

Yang Y-J, *et al. Open Heart* 2020; 7:e001139. doi: 10.1136/openhrt-2019-001139

Supplemental Data Part 5: Analysis of myocardial perfusion and viability by SPECT and PET

According to previous studies [7-11], myocardial perfusion was assessedby a dual-head SPECT (Siemens Medical, GER) 90 min after intravenously injecting a dose of 740 MBq technetium-99m sestamibi (99mTc-MIBI).Thirty-two projection images per 40 s in a 64×64 matrix were achieved by 180°-rotation arc from the 45- right and anterior sector to the 45- left and anterior sector. SPECT was reconstructed with Butterworth cut-off frequency of 0.45, with an order of 5 Quantitative analysis of imaging was performed by using Cedars quantitative perfusion SPECT software (QPS). The left ventricle was divided into 17 segments and scored from 0 to 4 automatically (0 = normal perfusion, 1 = mild perfusion decrease, 2 = moderate decrease, 3 = severe decrease, 4 = perfusion defect) according to the American Heart Association/American College of Cardiology recommendations [12]. The improvement of myocardial perfusion after cell infusion was defined as >1 score perfusion improvement in the segments pre-cell infusion compared with the relative segments post-cell infusion.

PET scans were performed within 2 days after SPECT. After an overnight fasting for at least 8 hours, an oral glucose dose of 50 g was given to the patients. Insulin was intravenously administered according to the blood glucose level. Fluorodeoxyglucose (FDG, 111–185 MBq) was administered intravenously when the blood glucose level was appropriate. Images were acquired 1 hour after tracer injection using a Biograph 64 PET/CT scanner (Siemens Medical Solutions, Knoxville, TN) equipped with

high-performance LSO PET crystals and a 64-slice CT. Images were collected and reconstructed using attenuation weighted-OSEM iterative reconstruction (8 subsets, 4 iterations). A non-gated, noncontrast-enhanced CT transmission scanning (140 kV, 35 mA) was performed for attenuation correction and anatomical localization preceded the PET scan.

After reconstruction, FDG uptake in left ventricle was quantitatively analyzed by QPS software in the same workstation as perfusion analysis. Areas of scar (perfusion–metabolism match) or hibernated myocardium (perfusion–metabolism mismatch) were assessed by two experienced nuclear medicine experts blinded to the present trial. The segmental tracer activity was calculated as a percentage of uptake of the segment with maximal uptake (normalization to the 100% perfusion maximum) and categorized on a 4-point scale: 1 denoting tracer activity of >75%; 2 denoting tracer activity of 50% to 75%; 3 denoting tracer activity of 25% to 49%; and 4 denoting tracer activity of <25%. Metabolic improvement was defined as a significant fill-in (>10%) of FDG defects was observed on the post-cell infusion images compared with baseline [13-15].

Supplemental Data Part 6: Sample size calculation

In a 2x2 factorial design trial, multiplicity issue should be considered to avoid the type I error inflation. Our detail considerations were as follow.

The present trial had three research hypotheses, the first one is to verify that bone marrow-mononuclear cells (MNC) can enhance LVEF of AMI patients; the second one is that intensive atorvastatin (IA) can increase LVEF of AMI patients than routine atorvastatin (RA); the third one is that IA can coordinate with MNC to further increase LVEF. We treated the above hypotheses in a hierarchical manner. The separate comparison of MNC and IA were tested at the first tier. In this tier, significance level was 0.025 (1-sided) for each comparison. By this way, the overall type I error could be controlled at 0.05. If any of the hypotheses in the first tier was significant, the last one assumption (IA + MNC vs. IA+Placebo) would be tested at a 2-sided 0.05 alpha level. Because the above sequential test process, it would have no impact on the overall type I error. These settings of significance level were incorporated into the sample size calculation procedure.

For the first hypothesis, according to the previous studies [Grajek S, et al. Eur Heart J. 2010; 31(6): 691-702. Traverse JH, et al. JAMA. 2011;306(19):2110-9.], LVEF at 6 months after AMI increases by 1% ± 3% on average compared to the baseline without MNC implantation, however, MNC transplantation can increase LVEF by 4% ± 5%. When the statistical significance level is taken as 1-sided 0.025 and the power as 80%, the minimal sample size of each group is calculated as 30 cases. For the second

research hypothesis, according to the previous report [Kim EK, et al. J Korean Med Sci. 2015. 30(4): 435-41], IA can increase LVEF by 3% ± 3% compared with RA. When the statistical significance level is 1-sided 0.025 and the power is 80%, the minimal sample size is 36 for each group. For the third hypothesis, according to our previous studies [Yang YJ, et al. European Heart Journal. 2008, 29(12):1578-90], it is estimated that the IA + MNC transplantation group can enhance LVEF of AMI patients by 9% ± 5%. When the statistical significance level is taken as 1-sided 0.025 and the power as 80%, the minimal sample size of each group is 16 cases. Combined with the sample size determined according to the statistical principle, and considering the randomization scheme and the possible maximum drop rate as 20%, this study plans to enroll 100 subjects who are allocated into group RA+Placebo, RA+MNCs, IA+Placebo, and IA+MNCs (25 cases in each group) according to the proportion of 1:1:1:1. In fact, according to the current design, this trial will provide more than 80% assurance to prove the expected research hypotheses.

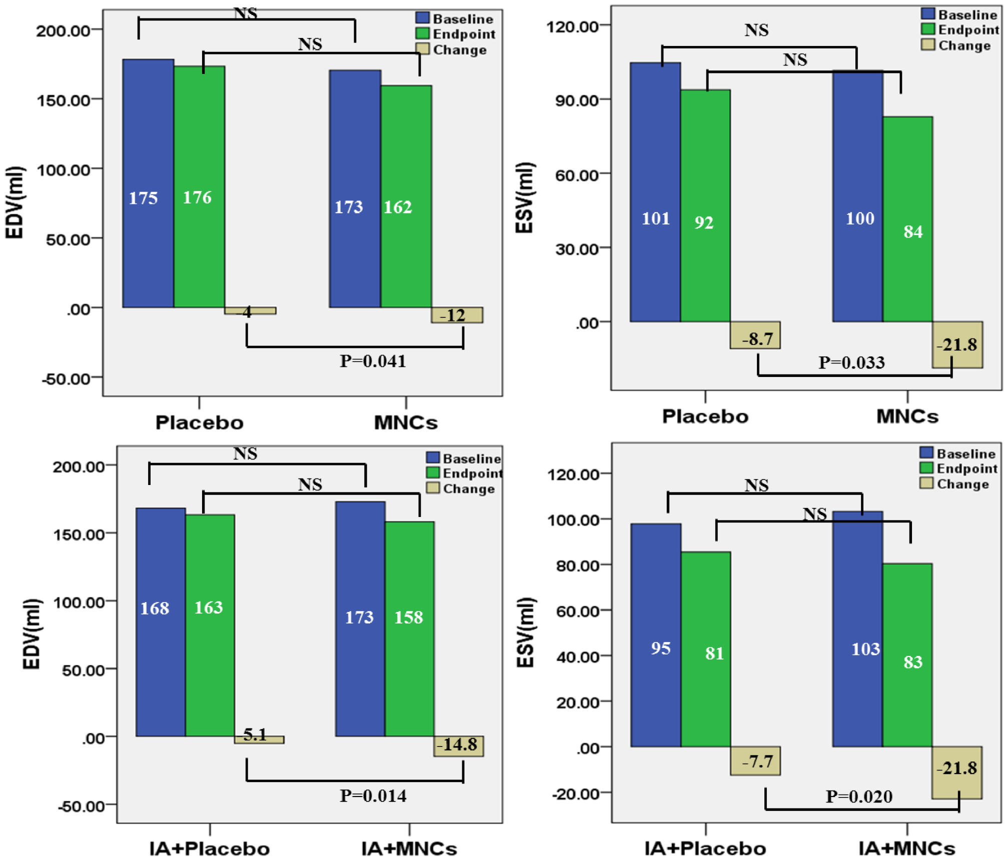

Supplemental Figure 1. The reductions in LV volumes with 2DE after MNCs transplants denoting LV remodeling attenuation

2DE: two-dimensional echocardiography; EDV:end-diastolic volume; ESV:end-systolic volume; IA: intensive atorvastatin; LV: left ventricle; MNCs: mononuclear cells; RA: regular atorvastatin

Supplemental Table 1. Baseline comparison between 24 patients lost to follow-up and 76 ones completing endpoint assessments

	**Patients completing endpoint assessments (n=76)**	**Patients lost to follow-up (n=24)**	***P value***
Male (%)	68(89.5)	23(95.8)	0.649
Age (years)	52.9±11.5	56.3±11.9	0.612
BMI (Kg/m^2)	25.1±5.6	25.6±4.3	0.784
Killip class	1.87±1.32	1.65±0.94	0.311
IABP (%)	7(9.2)	2(8.3)	0.659
Hypertension (%)	38(50.0)	14(58.3)	0.225
Diabetes (%)	17(22.4)	5(20.8)	0.203
Hyperlipidemia (%)	26(34.2)	9(37.5)	0.192
Stroke(%)	1(1.3)	1(4.1)	0.103
Smoking(%)	53(69.7)	18(75.0)	0.076
CHD family history(%)	11(14.5)	3(12.5)	0.233
Previous MI(%)	6(7.9)	2(8.3)	0.655
Previous coronary revascularization(%)	3(3.9)	1(4.2)	0.719
SBP(mmHg)	115.3±19.2	120.1±15.4	0.628
HR(bpm)	79.5±18.6	79.1±16.5	0.733

Yang Y-J, *et al. Open Heart* 2020; 7:e001139. doi: 10.1136/openhrt-2019-001139

ALT(IU/L)	61.5±39.2	47.3±30.6	0.478
Scr(umol/L)	82.8±15.1	88.5±18.2	0.827
HbA1c(%)	6.2±1.1	6.6±1.3	0.895
WBC($*10^9$/L)	8.8±2.9	9.3±1.8	0.859
HCT(%)	43.3±6.1	39.8±5.7	0.462
HGB(g/L)	143.6±17.1	139.5±15.8	0.489
PLT($*10^9$/L)	258.1±98.6	221.4±94.3	0.724
Medications			
Aspirin(%)	76(100)	24(100)	NS
Clopidogrel(%)	76(100)	24(100)	NS
Beta-blockers(%)	70(92.1)	21(87.5)	0.867
RAASi(%)	56(73.7)	20(83.3)	0.459
Spirolactone(%)	50(65.8)	16(66.7)	0.872
Diuretics(%)	55(72.4)	18(75.0)	0.856

ALT: alanine aminotransferase; BMI: body mass index; HbA1c: glycosylated hemoglobin; HCT: Hematocrit; HGB: Hemoglobin; HR: heart rate; IA: intensive aotrvastain; IABP: intra-aortic balloon pump; MNCs: mononuclear cells; PLT: platelet; RA: regular atorvastatin; RAASi: renin angiotensin aldosterone system inhibitor; SBP: systolic blood pressure; Scr: Serum creatinine; WBC: white blood cell;

Yang Y-J, *et al. Open Heart* 2020; 7:e001139. doi: 10.1136/openhrt-2019-001139

Supplemental Table 2. The properties of coronary lesions and intervention

	RA+Placebo	RA+MNCs	IA+Placebo	IA+MNCs	*P value*
Emergent PCI or thrombolysis(%)	5(26.3)	5(25.0)	8(47.1)	10(50.0)	0.225
LAD patency at implant(%)	8(42.1)	9(45.0)	10(58.8)	12(60.0)	0.272
LAD total occlusion at implant (%)	11(57.9)	11(55.0)	7(41.2)	8(40.0)	0.610
LAD lesion only(%)	7(36.8)	10(50.0)	6(35.3)	12(60.0)	0.386
Dual vessel lesions(%)	9(47.4)	8(40.0)	5(29.4)	3(15.0)	0.154
Triple vessel lesions(%)	3(15.8)	1(5.0)	5(29.4)	5(25.0)	0.215
LM lesion(%)	1(5.3)	1(5.0)	3(17.6)	1(5.0)	0.525
Stent implant(%)	18(94.7)	18(90.0)	14(82.4)	18(90.0)	0.688
Complete revascularization(%)	9(47.4)	10(50.0)	8(47.1)	9(45.0)	0.992
Duration from symptom onset to MNC infusion (d)	24.0±16.7	27.8±19.6	25.6±13.8	28.7±19.1	0.866

LAD: left anterior descending; LM: left main; MNCs: mononuclear cells;PCI: percutaneous coronary intervention;

Yang Y-J, *et al. Open Heart* 2020; 7:e001139. doi: 10.1136/openhrt-2019-001139

Supplemental Table 3. Blood lipid profile at baseline and one-year endpoint

	Total RA (n=39)	Total IA (n=37)	Total Placebo (n=36)	Total MNCs (n=40)
**TG(mmol/L)**				
Baseline	2.07±1.50	2.05±1.37	2.16±1.58	1.96±1.29
Endpoint	1.55±0.68	1.60±0.98	1.74±1.05	1.42±0.53
Adjusted Difference(95%CI) #	-0.52 (-1.06, -0.14)	-0.45 (-0.90, -0.07)	-0.42 (-0.95, -0.01)	-0.54 (-0.99, -0.19)
P value	0.821		0.695	
**TC(mmol/L)**				
Baseline	4.45±1.00	4.75±1.26	4.87±1.19	4.35±1.04
Endpoint	3.72±0.81	3.72±1.02	3.91±0.80	3.55±0.99
Adjusted Difference(95%CI) #	-0.72 (-1.00, -0.44)	-1.03 (-1.44, -0.67)	-0.96 (-1.27,-0.63)	-0.80 (-1.16, -0.47)
P value	0.203		0.512	
**LDL-C(mmol/L)**				
Baseline	2.41±0.78	2.85±0.90	2.78±0.87	2.44±0.83
Endpoint	2.09±0.78	2.08±0.75	2.19±0.71	2.00±0.80
Adjusted Difference(95%CI) #	-0.32 (-0.51,-0.18)	-0.78 (-0.97, -0.59)	-0.59 (-0.73, -0.37)	-0.45 (-0.69, -0.28)
P value	0.013		0.422	
**HDL-C(mmol/L)**				
Baseline	1.01±0.25	0.95±0.29	0.97±0.26	1.00±0.28
Endpoint	1.03±0.22	1.06±0.25	1.05±.019	1.04±0.27
Adjusted Difference(95%CI) #	0.02 (-0.15,0.11)	0.05 (-0.20,0.23)	0.06 (0.41,1.02)	0.04 (0.08,0.71)
P value		0.145		0.378

	Total RA（n=39）		Total IA（n=37）	
	**RA+Placebo (n=19)**	**RA+MNCs (n=20)**	**IA+Placebo (n=17)**	**IA+MNCs (n=20)**

**TG(mmol/L)**				
Baseline	2.11±1.68	2.02±1.35	2.22±1.51	1.90±1.25
Endpoint	1.71±0.82	1.39±0.47	1.78±1.28	1.44±0.60
Adjusted Difference(95%CI) #	-0.40 (-1.17,0.14)	-0.63 (-1.23,-0.17)	-0.44 (-1.07,0.10)	-0.46 (-1.12,0.04)
P value	0.634		0.967	
**TC(mmol/L)**				
Baseline	4.69±1.01	4.22±0.97	5.06±1.36	4.49±1.12
Endpoint	3.80±0.79	3.66±0.86	4.04±0.82	3.45±1.12
Adjusted Difference(95%CI) #	-0.90 (-1.33,-0.46)	-0.56 (-0.93,-0.20)	-1.03 (-1.50,-0.59)	-1.04 (-1.64,-0.51)
P value	0.269		0.985	
**LDL-C(mmol/L)**				
Baseline	2.61±0.75	2.22±0.78	3.04±0.99	2.71±0.83
Endpoint	2.13±0.70	2.06±0.86	2.28±0.73	1.92±0.75
Adjusted Difference(95%CI) #	-0.48 (-0.76,-0.18)	-0.16 (-0.33,-0.03)	-0.76 (-0.54,-0.25)	-0.79 (-1.12,-0.33)
P value	0.179		0.896	
**HDL-C(mmol/L)**				
Baseline	0.99±0.23	1.03±0.27	0.93±0.29	0.96±0.29
Endpoint	1.00±0.15	1.06±0.28	1.13±0.23	1.01±0.26
Adjusted Difference(95%CI) #	0.11 (0.05,0.21)	0.03 (0,0.17)	0.20 (0.09,0.31)	0.05 (0,0.25)
P value	0.857		0.209	

#values are median (95%CI), and adjusted for values at baseline and the other intervention

HDL-C:high density lipoprotein-cholesterol; IA: intensive aotrvastain; LDL-C:low density lipoprotein-cholesterol; MNCs: mononuclear cells;RA: regular atorvastatin; TC: total cholesterol; TG: triglyceride

Yang Y-J, *et al. Open Heart* 2020; 7:e001139. doi: 10.1136/openhrt-2019-001139

Supplemental Table 4 A. Comparison of LVEF by MRI after adding “missing values” of 24 patients lost to follow-up

	Total RA （n=50）	Total IA （n=50）	Total Placebo （n=50）	Total MNCs （n=50）
**LVEF**				
Baseline	35.3(32.1,40.5)	31.7(30.8,37.2)	32.4(31.2,34.8)	33.0(31.5,37.4)
Endpoint	42.4(40.1,45.3)	41.6(39.0,46.0)	36.5(37.0,43.3)	43.3(41.5,48.1)*
Adjusted Difference# (95%CI)	5.4(3.6,8.5)	8.2(6.4,10.7)	3.5(2.1,8.0)	8.6(6.4,11.2)
P value	0.085		0.026	

#values are median (95%CI) , and adjusted for values at baseline and the other intervention;

* endpoint values in total MNCs group *vs* total Placebo group(P=0.013);

Table 4 B. Comparison of LVEF between ATV groups after adding “missing values” of 24 patients lost to follow-up

	Total RA（n=50）		Total IA （n=50）	
	**RA+Placebo (n=25)**	**RA+MNCs (n=25)**	**IA+Placebo (n=25)**	**IA+MNCs (n=25)**
**LVEF**				
Baseline	35.2(27.5,38.1)	34.8(32.2,36.9)	33.1(29.9,35.0)	31.8(29.2,36.5)
Endpoint	40.3(34.4,43.2)	43.5(39.2,46.4)	36.9(33.5,40.7)	43.4(40.5,50.0)*
Adjusted Difference# (95%CI)	2.9(0.3,8.1)	5.8(4.1,8.9)	3.3(2.0,8.1)	9.5(7.7,16.5)
P value	0. 751		0.005	

#values are median (95%CI), and adjusted for values at baseline and the other intervention;

* the comparison of endpoint values in IA+ MNCs group *vs.* IA+Placebo group(P=0.001);

ATV: atorvastatin; IA: intensive atorvastatin; LVEF: left ventricular ejection fraction; MNCs: mononuclear cells; RA: regular atorvastatin.

Yang Y-J, *et al. Open Heart* 2020; 7:e001139. doi: 10.1136/openhrt-2019-001139

Supplemental Table 5.The structural and functional changes of left ventricle with 2DE after MNCs therapy

	Total RA （n=39）	Total IA （n=37）	Total Placebo （n=36）	Total MNCs （n=40）
**LVEF(%)**				
Baseline	43(40,45)	40(40,45)	41(40,45)	40(40,45)
Endpoint	45(43,50)	50(46,53)	47(44,50)	49(45,54)
Adjusted Difference#(95%CI)	5(2,7)	8(5,10)	4(2,6)	9(5,10)
P value	0.156		0.022	
**EDV(ml)**				
Baseline	174(169,180)	175(167,180)	175(170,181)	173(164,180)
Endpoint	170(160,177)	167(153,175)	176(164,184)	162(153,170)
Adjusted Difference# (95%CI)	-6.0(-10.0,-3.0)	-11.0(-14.0,-5.0)	-4.0(-7.0,-3.0)	-12.0(-17.0,-8.0)
P value	0.147		0.041	
**ESV(ml)**				
Baseline	100(95,110)	100(98,105)	101(98,105)	100(94,108)
Endpoint	92(86,99)	83(68,90)	92(85,99)	84(69,90)
Adjusted Difference# (95%CI)	-11.0(-20.2,-6.7)	-19.8(-24.4,-10.4)	-8.7(-13.8,-6.4)	-21.8(-25.3,-14.5)
P value	0.113		0.033	

	Total RA（n=39）		Total IA（n=37）	
	**RA+Placebo (n=19)**	**RA+MNCs (n=20)**	**IA+Placebo (n=17)**	**IA+MNCs (n=20)**
**LVEF(%)**				
Baseline	41(40,45)	42(37,45)	42(37,45)	40(39,45)
Endpoint	45(43,50)	46(42,55)	49(43,53)	50(46,54)
Adjusted Difference# (95%CI)	3(2,7)	6(0,14)	4(0,10)	10(8,12)
P value	0.188		0.045	
**EDV(ml)**				
Baseline	187(176,201)	167(157,179)	168(155,180)	173(163,184)
Endpoint	182(171,197)	161(147,175)§	163(147,177)	158(145,171)
Adjusted Difference#	-4.7(-7.3,-1.8)	-7.2(-14.5,0.6)	-5.1(-9.4,-0.9)	-14.8(-20.2,-8.9)

(95%CI)				
P value	0.578		0.014	
**ESV(ml)**				
Baseline	103(99,104)	95(90,111)	95(90,111)	103(99,114)
Endpoint	92(90,100)	85(65,102)	81(67,105)	83（68,89)
Adjusted Difference#(95%CI)	-10.0(-18.5,-6.7)	-19.4(-25.3,3.3)	-7.7(-24.4,-1.8)	-21.8(-29.4,-18.7)
P value	0. 381		0.020	

#values are median (95%CI) , and adjusted for values at baseline and the other intervention;

§the comparison of endpoint values in RA+ MNCs group *vs* RA+Placebo group(P=0.033)

2DE:two-dimensional echocardiography; EDV: end-diastolic volume; ESV: end-systolic volum; IA: intensive atorvastatin; LVEF: left ventricular ejection fraction; MNCs: mononuclear cells;RA: regular atorvastatin;

Supplemental Table 6. Myocardial perfusion with SPECT after MNCs transplantation

	Total RA （n=39）	Total IA （n=37）	Total Placebo （n=36）	Total MNCs （n=40）
**Perfusion Defect(%)**				
Baseline	34.2(31,37.3)	32(29,35)	33.5(30.3,36.7)	32.8(30.1,35.5)
Endpoint	32.1(29,35.1)	29.5(26.7,32.5)	31.4(28.3,34.5)	30.3(27.7,32.9)
Adjusted Difference#(95%CI)	-2.1(-2.4,-1.8)	-2.5(-2.8,-2.1)	-2.1(-2.3,-1.8)	-2.5(-2.9,-2.1)
P value	0.541		0.772	
**SRS**				
Baseline	22.2±6.9	20.9±6.5	21.7±7.3	21.4±6.3
Endpoint	21.7±6.9	20.1±6.5	21.3±7.4	20.6±6.0
Adjusted Difference# (95%CI)	-0.40 (-0.67,-0.17)	-0.41 (-0.70,-0.18)	-0.24 (-0.50,-0.07)	-0.57 (-0.88,-0.31)
P value	0.810		0.078	

	Total RA（n=39）		Total IA（n=37）	
	**RA+Placebo (n=19)**	**RA+MNCs (n=20)**	**IA+Placebo (n=17)**	**IA+MNCs (n=20)**
**Perfusion Defect(%)**				
Baseline	34.7(29.9,40.3)	33.8(29.9,37.9)	32.3(27.8,37.1)	31.8(28.1,35.8)
Endpoint	32.8(27.9,38.2)	31.5(27.7,35.4)	30.1(25.6,34.8)	29.1(25.6,32.8)
Adjusted Difference# (95%CI)	-1.9(-2.4,-1.6)	-2.3(-2.8,-1.8)	-2.2(-2.5,-1.9)	-2.7(-3.3,-2.0)
P value	0.285		0.288	
**SRS**				
Baseline	22.4±7.5	22.1±6.6	21.0±7.2	20.8±6.1
Endpoint	22.2±7.5	21.4±6.4	20.5±7.5	19.9±5.6
Adjusted Difference# (95%CI)	-0.19 (-0.56,0)	-0.64 (-1.15,-0.20)	-0.31 (-0.73,0)	-0.50 (-0.93,-0.21)
P value	0.101		0.390	

#values are median (95%CI) , and adjusted for values at baseline and the other intervention;

IA: intensive atorvastatin; MNCs: mononuclear cells;RA: regular atorvastatin; SRS: summed rest score

Supplemental Table 7. Biomarkers at baseline and endpoint

	Total RA （n=39）	Total IA （n=37）	Total Placebo （n=36）	Total MNCs （n=40）
**NT-proBNP(pg/ml)**				
Baseline	1716.7±135.5	1801.5±177.0	1703.8±132.8	1801.8±170.7
Endpoint	754.2±82.2	590.0±124.3	914.2±127.8	669.9±55.6
Adjusted difference# (95%CI)	-962.4±155.8	-1211.5±179.8	-789.6±143.3	-1340.2±175.5
P value	0.316		0.023	
**hs-CRP(mg/L)**				
Baseline	7.7±5.2	7.8±4.6	8.3±4.7	7.2±5.1
Endpoint	2.5±2.2	1.3±1.0§	1.9±1.6	2.5±1.8
Adjusted difference# (95%CI)	5.9(5.0,6.8)	6.8(5.9,7.7)	6.1(5.2,7.1)	6.7(5.8,7.6)
P value	0.369		0.392	
**Endothelin(pmol/L)**				
Baseline	0.74 (0.55, 0.86)	0.67 (0.52, 0.89)	0.65 (0.54, 0.84)	0.82 (0.54, 1.20)
Endpoint	0.50 (0.43, 0.72)	0.54 (0.40, 0.74)	0.54 (0.39, 0.81)	0.51 (0.43, 0.73)
Adjusted difference# (95%CI)	0.09 (-0.09, 0.40)	0.11 (-0.14, 0.55)	0.06 (-0.10, 0.33)	0.10 (-0.13, 0.63)
P value		0.950		0.799

	Total RA（n=39）		Total IA（n=37）	
	**RA+Placebo (n=19)**	**RA+MNCs (n=20)**	**IA+Placebo (n=17)**	**IA+MNCs (n=20)**
**NT-proBNP(pg/ml)**				
Baseline	1644.3±163.0	1776.2±201.4	1767.8±222.3	1841.3±288.5
Endpoint	921.2±170.5	616.7±68.5	906.6±190.1	215.8±18.0*
Difference	-723.1±238.3	-1159.5±206.6	-861.2±172.3	-1652.5±289.3
P value	0.190		0.034	
**hs-CRP(mg/L)**				

Baseline	8.13±4.81	7.19±5.67	9.10±4.66	7.36±4.61
Endpoint	2.17±2.13	2.24±2.06	1.00±0.89	1.24±0.62
Adjusted Difference# (95%CI)	-6.07 (-5.47,-7.08)	-4.86 (-4.03,-5.68)	-8.06 (-7.17,-8.98)	-6.13 (-5.43,-7.01)
P value	0.563		0.370	
**Endothelin(pmol/L)**				
Baseline	0.73 (0.51, 0.80)	0.71 (0.57, 0.99)	0.68 (0.59, 0.86)	0.81 (0.62, 1.00)
Endpoint	0.53 (0.41, 0.68)	0.50 (0.38, 0.65)	0.52 (0.37, 0.76)	0.49 (0.33, 0.61)
Adjusted Difference(95%CI) #	-0.09 (-0.06, -0.31)	-0.13 (-0.16, -0.45)	0.07 (-0.45,0.19)	-0.20 (-0.61,0.22)
P value	0.180		0.046	

#values are median (95%CI) , and adjusted for values at baseline and the other intervention;

§ the endpoint values in total RA *vs* total IA group (P=0.001);

*the endpoint values in total MNCs group ***vs*** total Placebo group (P=0.005)

hs-CRP: high sensitivity C reactive protein; IA: intensive atorvastatin; MNCs: mononuclear cells;NT-proBNP:amino-terminal pro-B-type natriuretic peptide; RA: regular atorvastatin

Supplemental Table 8. MACEs after MNC therapy

	Total RA （n=39）	Total IA （n=37）	Total Placebo （n=36）	Total MNCs （n=40）
**Death**	0	0	0	0
P value	NS		NS	
**Recurrent MI**	0	0	0	0
P value	NS		NS	
**TVR**	0	0	0	0
P value	NS		NS	
**New arrhythmia**	0	0	0	0
P value	NS		NS	

	Total RA（n=39）		Total IA（n=37）	
	RA+Placebo (n=19)	RA+MNCs (n=20)	IA+Placebo (n=17)	IA+MNCs (n=20)
**Death**	0	0	0	0
P value	NS		NS	
**Recurrent MI**	0	0	0	0
P value	NS		NS	
**TVR**	0	0	0	0
P value	NS		NS	
**New arrhythmia**	0	0	0	0
P value	NS		NS	

IA: intensive atorvastatin; MI: myocardial infarction; MNCs: mononuclear cells;RA: regular atorvastatin; TVR: target vascular revascularization

Yang Y-J, *et al. Open Heart* 2020; 7:e001139. doi: 10.1136/openhrt-2019-001139

Supplemental References

[1] Qian H, Yang Y, Huang J, et al. Intracoronary delivery of autologous bone marrow mononuclear cells radiolabeled by 18F-fluoro-deoxy-glucose: tissue distribution and impact on post-infarct swine hearts. J Cell Biochem 2007;102:64-74.

[2] Yang YJ, Qian HY, Huang J, et al. Atorvastatin treatment improved post-infarction microenvironments to facilitate the survivals and activities of implanted mesenchymal stem cells in swine hearts with acute myocardial infarction and reperfusion. Eur Heart J 2008, 29:1578-90.

[3] Yang YJ, Qian HY, Huang J,et al. Combined therapy with simvastatin and bone marrow-derived mesenchymal stem cells increases benefits in infarcted swine hearts. Arterioscler Thromb Vasc Biol 2009;29:2076-82.

[4] Assmus B, Urbich C, Aicher A, et al. HMG-CoA reductase inhibitors reduce senescence and increase proliferation of endothelial progenitor cells via regulation of cell cycle regulatory genes. Circ Res 2003;92:1049-55.

[5] Song L, Yang YJ, Dong QT, et al. Atorvastatin enhance efficacy of mesenchymal stem cells treatment for swine myocardial infarction via activation of nitric oxide synthase. PLoS One 2013; 8:e65702.

[6] Cao F, Sun D, Li C, et al. Long-termmyocardialfunctional improvement after autologousbonemarrowmononuclearcellstransplantation in patients with ST-segment elevationmyocardial infarction: 4 years follow-up. Eur Heart J 2009;30:1986-94.

[7] Choudry F, Hamshere S, Saunders N, et al. A randomized double-blind control study of early intra-coronary autologousbonemarrowcell infusion in acutemyocardial infarction: the REGENERATE-AMIclinical trial. Eur Heart J 2016;37:256-63.

[8] Assmus B, Urbich C, Aicher A, et al. HMG-CoA reductase inhibitors reduce senescence and increase proliferation of endothelial progenitor cells via regulation of cell cycle regulatory genes. Circ Res 2003;92:1049-55.

[9] Klocke FJ, Baird MG, Lorell BH, et al; American College of Cardiology; American Heart Association Task Force on Practice Guidelines; American Society for Nuclear Cardiology. ACC/AHA/ASNC guidelines for the clinical use of cardiac radionuclide imaging--executive summary: a report of the American College of Cardiology/American Heart Association Task Force on Practice Guidelines (ACC/AHA/ASNC Committee to Revise the 1995 Guidelines for the Clinical Use of Cardiac Radionuclide Imaging).Circulation. 2003;108(11):1404-18.

[10] Dilsizian V, Bacharach SL, Beanlands R, et al. ASNC imaging guidelines for nuclear cardiology procedures, PET myocardial perfusion and metabolism clinical imaging. J Nucl Cardiol 2009;16:651-80.

[11] Patila T, Lehtinen M, Vento A, et al. Autologous bone marrow mononuclear cell transplantation in ischemic heart failure: a prospective, controlled, randomized, double-blind study of cell transplantation combined with coronary bypass. J Heart Lung Transplant 2014; 33:567-74.

[12] Luger D, Lipinski MJ, Westman PC, et al. Intravenously Delivered Mesenchymal Stem Cells: Systemic Anti-Inflammatory Effects Improve Left Ventricular Dysfunction in Acute Myocardial Infarction and Ischemic Cardiomyopathy. Circ Res 2017;120:1598-613.

[13] Pasha Z, Wang Y, Sheikh R, et al. Preconditioning enhances cell survival and differentiation of stem cells during transplantation in infarcted myocardium. Cardiovasc Res 2008; 77: 134-42.

[14] Shafei AE, Ali MA, Ghanem HG, et al. Mesenchymal stem cell therapy: A promising cell-based therapy for treatment of myocardial infarction. J Gene Med

Yang Y-J, *et al. Open Heart* 2020; 7:e001139. doi: 10.1136/openhrt-2019-001139

2017; 19: e2995.

[15] Simari RD,Pepine CJ,Traverse JH,et al. Bone marrowmononuclearcell therapy for acutemyocardial infarction: a perspective from the cardiovascular cell therapy research network.Cir Res 2014;114:1564-68.
